# Risk factors for delirium in intensive care patients: a prospective cohort study

**DOI:** 10.1186/cc7892

**Published:** 2009-05-20

**Authors:** Bart Van Rompaey, Monique M Elseviers, Marieke J Schuurmans, Lillie M Shortridge-Baggett, Steven Truijen, Leo Bossaert

**Affiliations:** 1 University of Antwerp, Faculty of Medicine, Division of Nursing Science and Midwifery, Universiteitsplein 1, 2610 Wilrijk, Belgium; 2 Artesis University College of Antwerp, Department of Health Sciences, J. De Boeckstraat 10, 2170 Merksem, Belgium; 3 University of Professional Education Utrecht, Department of Healthcare, Bolognalaan 101, postbus 85182, 3508 AD Utrecht, The Netherlands; 4 Pace University, Lienhard School of Nursing, Lienhard Hall, Pleasantville, New York 10570, USA; 5 University Hospital of Antwerp, Intensive Care Department, Belgium; 6 University of Antwerp, Faculty of Medicine, Universiteitsplein 1, 2610 Wilrijk, Belgium

## Abstract

**Introduction:**

Delirium is a common complication in the intensive care unit. The attention of researchers has shifted from the treatment to the prevention of the syndrome necessitating the study of associated risk factors.

**Methods:**

In a multicenter study at one university hospital, two community hospitals and one private hospital, all consecutive newly admitted adult patients were screened and included when reaching a Glasgow Coma Scale greater than 10. Nurse researchers assessed the patients for delirium using the NEECHAM Confusion Scale. Risk factors covered four domains: patient characteristics, chronic pathology, acute illness and environmental factors. Odds ratios were calculated using univariate binary logistic regression.

**Results:**

A total population of 523 patients was screened for delirium. The studied factors showed some variability according to the participating hospitals. The overall delirium incidence was 30%. Age was not a significant risk factor. Intensive smoking (OR 2.04), daily use of more than three units of alcohol (OR 3.23), and living alone at home (OR 1.94), however, contributed to the development of delirium. In the domain of chronic pathology a pre-existing cognitive impairment was an important risk factor (OR 2.41). In the domain of factors related to acute illness the use of drains, tubes and catheters, acute illness scores, the use of psychoactive medication, a preceding period of sedation, coma or mechanical ventilation showed significant risk with odds ratios ranging from 1.04 to 13.66. Environmental risk factors were isolation (OR 2.89), the absence of visit (OR 3.73), the absence of visible daylight (OR 2.39), a transfer from another ward (OR 1.98), and the use of physical restraints (OR 33.84).

**Conclusions:**

This multicenter study indicated risk factors for delirium in the intensive care unit related to patient characteristics, chronic pathology, acute illness, and the environment. Particularly among those related to the acute illness and the environment, several factors are suitable for preventive action.

## Introduction

Delirium is a common complication in the intensive care unit. The acute syndrome, caused by a disturbance of the cognitive processes in the brain, is characterized by a reduced ability to focus, sustain or shift attention, disorganized thinking or a changed level in consciousness. The pathophysiology is based on different neurochemical processes induced by a physical cause. Multiple factors seem to stimulate abnormal processes in the human brain [1].

Despite the international efforts, no evidence-based treatment or management of delirium in the intensive care unit has been established [2]. Proposed guidelines or an existing delirium protocol might not be available or known by the intensive care staff [3]. Nurses and physicians should assess patients for delirium. A standardized screening for delirium, however, is not common in most intensive care units.

The attention of researchers has shifted from the treatment to the prevention of the syndrome necessitating the study of associated risk factors. Delirium is never caused by a single factor, but is always the consequence of multiple factors. Inouye and colleagues [4] conceived a risk model for patients outside the intensive care unit based on predisposing and precipitating factors. Predisposing factors are patient dependent or related to chronic pathology. These factors are limited or not modifiable. Precipitating factors are related to the acute illness or the environment. In the intensive care unit current illness and aggressive treatment generate different impacts.

More than 60 variables have been studied for their relation with delirium in the general hospital population. A patient encountering three or more of these factors has a 60% increased risk for the development of delirium [4,5]. Ely and colleagues [6] stated that a patient in the intensive care unit accumulates 10 or more of these factors. As not all patients in the intensive care unit may develop delirium, it seems obvious that not all factors studied in general patients or elderly may be extrapolated to the intensive care patient. Therefore, each factor must be studied in the context of the intensive care unit. Earlier research on risk factors for delirium in the intensive care unit, using different methods and populations, showed sometimes conflicting results [7-11]. Additionally, environmental factors are poorly studied in the intensive care unit.

An intervention on relevant factors could influence the incidence of delirium in the intensive care unit. To prevent delirium, precipitating factors are more modifiable than predisposing factors. This research studied factors related to patient characteristics, chronic pathology, acute illness, and the environment for their contribution to the development of delirium in the intensive care patient.

## Materials and methods

### Study design

A prospective cohort study included patients at different locations based on a single protocol. All consecutive patients in the intensive care units of four hospitals, two community hospitals, one private hospital and one university hospital, were screened for delirium and associated risk factors by trained nurse researchers under supervision of the first author.

All consecutive patients with a minimum age of 18 years and a stay of at least 24 hours in the intensive care unit were included when reaching a Glasgow Coma Scale of at least 10. None of the patients was intubated at the time of the assessments. All patients were able to communicate with the nurse researchers. Patients or their relatives gave informed consent to the study. The ethical board of the hospitals approved the study.

The data were obtained in a first period of data collection from January to April 2007 in the university hospital and in a second period from January to April 2008 in separate studies in the community hospitals, the private hospital, and the university hospital again. The separate studies used the same methodology and all nurse researchers used the same standardized list to screen possible factors. Not all factors, however, were scored identically at the different locations. Non-identical data were deleted from the database. One hospital did not report on all factors. Therefore, the studied factors showed some variability according to the participating hospitals (Table 1). For the non-delirious patients the highest score of the possible risk factors of the entire observation period was selected. For delirious patients the highest score before the onset of delirium was registered.

**Table 1:** Number of the factors scored with indication of the site where the factor was included

	n	Community hospital (n = 210)	Private hospital (n = 123)	University hospital (n = 190)
domain patient characteristics				

age in years (mean, SD)	523	X	X	X
age more than 65 years	523	X	X	X
gender masculine	523	X	X	X
living single at home	182	X		X
units of alcohol per day	230	X		X
daily use of alcohol	496	X	X	X
daily use of more than three units of alcohol	230	X		X
number of cigarettes per day	221	X		X
daily smoking	519	X	X	X
daily smoking of more than 10 cigarettes	217	X		X

domain chronic pathology				

predisposing cognitive impairment	384	X	X	X
predisposing cardiac disease	265	X		X
predisposing pulmonary disease	262	X		X

**domain acute illness**				

length of stay in the ICU before inclusion	523	X	X	X
length of stay in the ICU before inclusion >1 day	523	X	X	X
length of stay in the ICU before inclusion >2 days	523	X	X	X
admission for internal medicine	523	X	X	X
high risk of mortality (SAPS >40; APACHE > 24)	212	X		X
APACHE II	120	X		X
SAPS II	108	X		
highest TISS 28 score	179	X		X
mean TISS 28	179	X		X
TISS 28 cut off 30 (318 minutes)	279	X		X
psychoactive medication	424	X	X	X
benzodiazepine	283	X	*X(low response)*	X
morphine	287	X	*X(low response)*	X
sedation	228	X	*X(low response)*	X
endotracheal tube or tracheastomy	390	X		X
gastric tube	395	X		X
bladder catheter	400	X		X
arterial catheter	398	X		X
number of perfusions	400	X		X
more than three perfusions	398	X		X
number of vascular catheters	400	X		X
no normal food	395	X		X
fever	397	X		X

**domain environmental factors**				

admission via emergency room	377	X		X
admission via transfer	377	X		X
open room in intensive care	508	X	X	X
isolation	523	X	X	X
no visible daylight	523	X	X	X
no clock present or visible	523	X	X	X
number of visitors	256	X	X	X
no visit	269	X	X	X
physical restraints	292	X		X

APACHE = Acute Physiology And Chronic Health Evaluation; ICU = intensive care unit; SAPS = Simplified Acute Physiology Score; SD = standard deviation; TISS 28 = The Therapeutic Intervention Scoring System-28.

The databases were joined based on depersonalised coded data. Patients from the different units were included using the same criteria resulting in a mixed intensive care population.

### Delirium assessment

All patients were screened for delirium using the Neelon and Champagne Confusion Scale [12-14]. Earlier research indicated this scale as a valuable tool for screening delirium in the intensive care unit by trained nurses [15]. This tool uses standard nursing observations to rate the patient on a 0 to 30 scale. A score 0 to 19 indicates delirium, whereas scores between 20 and 24 indicate mild or beginning confusion, 25 to 26 indicate a patient at risk for confusion and 27 to 30 indicates a normal patient.

### Assessment of the risk factors

**Figure 1:**
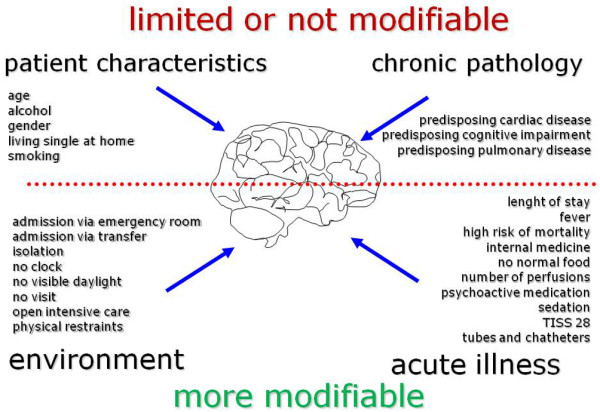
Four domains of risk factors for intensive care delirium.  TISS 28 = The Therapeutic Intervention Scoring System-28.

Factors were grouped into four domains based on the predisposing and precipitating model of Inouye and colleagues [4], the remarks of Ely [16], and the experience of intensive care staff: patient characteristics, chronic pathology, acute illness, and environmental factors (Figure 1). The first two domains contain predisposing or achieved factors being less modifiable through preventive actions. The last two domains apply to the current situation and are probably more modifiable to reduce the incidence of intensive care delirium.

In the domain of the patient characteristics, age, gender, and daily smoking or alcohol usage habits were scored in almost all patients. Patients or their relative often reported inexact values for number of cigarettes or units of alcohol used daily. These data were not reported by the private hospital. At two locations, the community hospital and one study in the university hospital, supplementary data on the social and matrimonial status, profession, and education of the patient were obtained.

In the domain of the chronic illness, the main focus was on a pre-existing cognitive impairment. This item was scored as positive when an established diagnosis of dementia was recorded in the medical record of the patient. All hospitals, except the private hospital, mentioned chronic cardiac or pulmonary diseases reported in the patient's record.

In the domain of the acute illness, factors were studied relating to the current diagnosis or treatment. All patients could be classified as either a surgical or an internal medicine patient. As patients were included at the time they scored a Glasgow Coma Scale of 10 or more, the length of stay in the intensive care unit before inclusion was observed as an indicator for coma or induced coma. Fever, temperature over 38.5°C, nutrition, and the use of drains, tubes, and catheters were observed at four locations. The number of infusions was transformed in a dichotomous factor 'more than three infusions' based on the relative risk for 'more than three medications added' (relative risk (RR), 2.9; 95% confidence interval (CI), 1.6 to 5.4) described by Inouye and colleagues [4]. The admittance of psychoactive medication before delirium, including the use of morphine and benzodiazepines, was scored in all studies. A risk of mortality score, the Simplified Acute Physiology Score (SAPS II) [17] or the Acute Physiology And Chronic Health Evaluation (APACHE II) [18], was observed in the university hospital and one community hospital. The two scores were transformed in a binary scoring factor 'high risk for mortality' indicating an APACHE II of at least 24 or a SAPS II score of at least 40. The Therapeutic Intervention Scoring System-28 (TISS 28) was scored in patients at the same locations [19]. A cut-off value of 30 was used indicating a nursing time workload of 318 minutes during each nursing shift.

Factors from the fourth domain relate to architectonical items or the interaction between the patient and the environment. Admission characteristics, the presence of visible daylight, the presence of a visible clock, and the architectonical structure, e.g. an open space with several patients or a closed room, were scored at all locations. Three studies reported on the use of physical restraints and relatives visiting the patient.

### Statistical approach and analysis

Continuous or categorical data were transformed to factors with a binary score. Cut-off values were based on literature or the variance of the data. For the non-delirious patients the most severe score of the possible risk factors of the entire observation period was selected. For delirious patients the most severe score before the onset of delirium was taken for the analysis.

The tables present the data for delirious and non-delirious patients. For each factor, the number of patients in both groups is mentioned. Continuous data are presented using mean and standard deviation. Categorical data are presented in percentages indicating the prevalence of the factor in either the delirium or the non-delirium group. Differences between delirious and non-delirious patients were calculated using the independent t-sample test or the Pearson Chi-squared test where appropriate.

Odds ratios (OR) with a 95% CI were calculated for all factors using univariate binary logistic regression. To facilitate reading, the text does not mention the CI values. The tables presenting the risk factors of the different domains, however, show the OR and CI values. Only factors with a prevalence of 10% in the delirious group and with a significant increased risk for delirium after univariate analysis were used in a multivariate forward conditional (0.05) regression analysis. Factors showing a wide CI after univariate analysis were not used in the multivariate analysis. The Nagelkerke regression coefficient was used to explain the variation in delirium predicted by the factors in the different domains.

A level of significance of 0.05 was used for all analysis. All statistics were calculated using SPSS 16.0 ^® ^(SPSS inc., Chicago, Illinois, USA).

## Results

A total population of 523 patients was screened for delirium and associated risk factors (Table 2). The overall incidence of delirium was 30%. Of 155 delirious patients, 75% were delirious on the first day of inclusion, and more than 90% after the third day. The incidence in the community hospitals was higher than the incidence in the private hospital or the university hospital. The mean age was 64 years and most of the population was male. The surgical and internal patients are equally represented, but the participating hospitals showed some variety. Patients tended to stay longer in the intensive care unit of the community hospital, but the length of stay in the intensive care unit before inclusion was the same for all hospitals. More than 60% of the patients had an immediate inclusion in the study with regard to the protocol (24 hours after admission to the intensive care unit). After 48 hours of admission to the intensive care unit, almost 80% of the population was included.

**Table 2:** Baseline Characteristics

		Total population	Community hospital	Private hospital	University hospital	*P *value
N		523	210	123	190	

age in years	mean (range)	64 (19 to 90)	65 (19 to 90)	67 (26 to 87)	60 (20 to 90)	<0.001

gender	male	59%	61%	54%	62%	0.34

admission	surgery	49%	26%	73%	59%	<0.001

internal medicine	51%	74%	27%	41%		

length of stay in days	mean (range)	8 (1 to 68)	11 (2 to 68)	7 (2 to 43)	8 (1 to 54)	0.01

length of stay before inclusion in days	mean (range)	3.6 (1 to 63)	3.9 (1 to 63)	3.5(1 to 34)	3.2 (1 to 47)	0.62

						

NEECHAM	delirium	29.6%	38%	29%	21%	<0.001

early to mild confused	25.8%	23%	33%	24%		

at risk	19.7%	10%	21%	30%		

normal	24.9%	29%	17%	26%		

						

APACHE II	mean (range)	--------	15 (19 to 23)	--------	19 (7 to 47)	0.04

SAPS II	mean (range)	--------	31 (4 to 73)	--------	--------	

TISS 28	mean (range)	--------	34 (19 to 48)	--------	32 (17 to 49)	0.19

						

capacity of the intensive care units			25 beds	24 beds	34 beds	

*P *value for difference between groups was calculated with the independent samples t-test for continuous data and Chi squared for categorical data.

APACHE = Acute Physiology And Chronic Health Evaluation; NEECHAM = Neelon and Champagne Confusion Scale; SAPS = Simplified Acute Physiology Score; TISS 28 = The Therapeutic Intervention Scoring System-28.

### Factors related to patient characteristics

**Figure 2:**
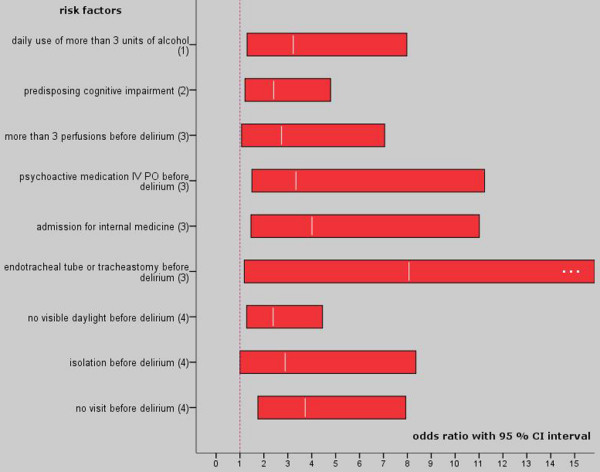
Multivariate risk factors for intensive care delirium.  Odds ratio with 95% confidence interval (CI), the number behind the factor indicates the domain: patients characteristics; chronic pathology; acute illness; and environment.

Neither age, age over 65 years, nor gender showed a relation to the onset of delirium in this study. Patients living alone at home had a higher risk of developing delirium (OR 1.94; Table 3). The use of alcohol was a significant risk factor for delirium when a patient consumed more than three units each day. Moreover, this factor showed a higher risk after multivariate analysis (OR 3.23; Figure 2). Each cigarette increased the risk for delirium, showing a significant OR for patients smoking 10 cigarettes or more each day (OR 2.04).

**Table 3:** Factors related to patient characteristics

	n		Mean (SD) or %			univariate	multivariate
	D	ND	D	ND	P*	OR (CI)	OR (CI)

age in years (mean, SD)	155	368	65.0 (16.4)	63.7 (14.6)	0.36	1.01 (0.99 to 1.02)	

age more than 65	91/155	202/368	55%	59%	0.24	1.17 (0.80 to 1.71)	

gender masculine	90/155	220/368	58%	60%	0.40	0.93 (0.64 to 1.36)	

living single at home	45/114	38/68	56%	40%	0.02	1.94 (1.06 to 3.57)	

units of alcohol per day	58	172	3.2 (5.2)	2.1 (3.9)	0.09	1.05 (0.99 to 1.12)	

daily use of alcohol	44/142	94/354	31%	27%	0.19	1.24 (0.81 to 1.90)	

daily use of more than three units of alcohol	21/58	32/172	36%	19%	0.01	2.48 (1.29 to 4.80)	3.23 (1.30 to 7.98)

number of cigarettes per day	46	175	11.4 (13.6)	6.4 (9.6)	0.02	1.04 (1.01 to 1.07)	

daily smoking	33/153	98/366	22%	27%	0.13	0.75 (0.48 to 1.18)	

daily smoking of more than 10 cigarettes	22/46	54/174	48%	31%	0.03	2.04 (1.05 to 3.95)	

Continuous variables are presented in number, mean and standard deviation (SD); categorical variables are presented in number per group and percentage.

* *P *value of difference in groups, calculated with independent samples t-test for continuous variables, with Chi squared for categorical variables.

CI = confidence interval; D = delirium group; ND = non-delirium group; OR = odds ratio.

### Factors related to chronic pathology

In the domain of chronic pathology only a predisposing cognitive impairment, indicating an established diagnosis of dementia, was a risk factor (Table 4).

**Table 4:** Factors related to chronic pathology

	n		%		P*	univariate	multivariate
	D	ND	D	ND		OR (CI)	OR (CI)

predisposing cognitive impairment	19/107	25/277	18%	9%	0.02	2.18 (1.14 to 4.14)	2.41 (1.21 to 4.79)

predisposing cardiac disease	36/72	112/193	50%	58%	0.15	0.72 (0.42 to 1.25)	

predisposing pulmonary disease	18/72	47/190	25%	25%	0.54	1.01 (0.54 to 1.90)	

Categorical variables are presented in number per group and percentage.

* *P *value of difference in groups, calculated with independent samples t-test for continuous variables, with Chi squared for categorical variables.

CI = confidence interval; D = delirium group; ND = non-delirium group; OR = odds ratio.

This factor remained significant after correction with the non-significant factors in the domain (OR 2.41; Figure 2). Pre-existing cardiac or pulmonary diseases were no risk factors in the studied cohort.

### Factors related to acute illness

The prevalence of abnormal blood values in the delirium group was too low to be considered in this study.

The length of stay in the intensive care unit before inclusion was shown to be a relevant factor in the onset of delirium. Based on the length of stay before inclusion as a risk factor, the risk for delirium increased by 26% each day (Table 5). Patients admitted for internal medicine had a higher risk of developing delirium than surgical patients, even after multivariate analysis (OR 4.01; Figure 2). The high risk of mortality score indicated that patients scoring an APACHE II higher than 24 or a SAPS II higher than 40 were at risk for delirium (OR 2.50). The TISS-28 score showed significant ORs in all calculations. The cut-off value of 30 was shown to be a relevant marker in the onset of delirium (OR 2.81). Yet, none of those scores for the intensive care unit shown it to be a risk factor after multivariate analysis (Table 5).

**Table 5:** Factors related to acute illness

	n		mean (SD) or %			univariate	multivariate
	D	ND	D	ND	P*	OR (CI)	OR (CI)

length of stay in the ICU before inclusion*	155	368	7.9 (11.5)	1.7 (2.3)	<0.001	1.26 (1.17 to 1.35)	

length of stay in the ICU before inclusion >1 day*	87/155	116/368	56%	32%	<0.001	2.78 (1.89 to 4.09)	

length of stay in the ICU before inclusion >2 days*	70/155	46/368	45%	13%	<0.001	5.77 (3.71 to 8.97)	

admission for internal medicine	91/155	175/368	48%	59%	0.013	1.57 (1.07 to 2.29)	4.01 (1.46 to 11.01)

high risk of mortality (SAPS >40; APACHE >24)	29/73	29/139	40%	21%	0.003	2.50 (1.31 to 4.66)	

APACHE II	33	87	19.7 (7.3)	18.6 (7.5)	0.47	1.02 (0.97 to 1.08)	

SAPS II	54	54	33.4 (12.6)	28.6 (10.7)	0.04	1.04 (1.01 to 1.08)	

highest TISS 28 score	88	191	34.9 (5.7)	31.9 (6.6)	<0.001	1.08 (1.04 to 1.13)	

mean TISS 28	88	191	30.8 (3.9)	29.1 (5.6)	0.004	1.07 (1.02 to 1.13)	

TISS 28 cut off 30 (318 minutes)	68/88	104/191	77%	55%	<0.001	2.81 (1.60 to 5.05)	

psychoactive medication	103/135	146/289	76%	51%	<0.001	3.15 (1.99 to 4.99)	3.34 (1.50 to 11.23)

benzodiazepine	18/68	24/215	27%	11%	0.003	2.89 (1.44 to 5.69)	

Morphine	24/70	54/217	34%	25%	0.09	1.58 (0.88 to 2.82)	

sedation	65/88	24/140	74%	17%	<0.001	13.66 (7.15 to 26.10)	

endotracheal tube or tracheastomy	27/118	11/272	23%	4%	<0.001	7.04 (3.36 to 14.76)	8.07 (1.18 to 55.06)

gastric tube	44/120	19/275	37%	7%	<0.001	7.80 (4.30 to 14.16)	

bladder catheter	115/120	227/280	96%	81%	<0.001	5.37 (2.09 to 13.80)	

arterial catheter	108/120	231/278	90%	83%	0.05	1.83 (0.93 to 3.59)	

number of perfusions	120	280	4.2 (2.0)	3.1 (1.7)	<0.001	1.35 (1.20 to 1.52)	

more than three perfusions	65/120	81/278	54%	29%	<0.001	2.87 (1.85 to 4.47)	2.74 (1.07 to 7.05)

number of vascular catheters	120	280	1.2 (0.5)	1.3 (0.6)	0.18	0.74 (0.47 to 1.17)	

no normal food	92/120	127/275	77%	46%	<0.001	3.83 (2.36 to 6.22)	

fever	10/119	16/278	8%	6%	0.222	1.50 (0.66 to 3.42)	

*: the only reason for later inclusion of patients was a score on the Glasgow Coma Scale below 10.

Continuous variables are presented in number, mean and standard deviation (SD); categorical variables are presented in number per group and percentage.

* *P *value of difference in groups, calculated with independent samples t-test for continuous variables, with Chi squared for categorical variables.

APACHE = Acute Physiology And Chronic Health Evaluation; CI = confidence interval; D = delirium group; ICU = intensive care unit; ND = non-delirium group; OR = odds ratio; SAPS = Simplified Acute Physiology Score; TISS 28 = The Therapeutic Intervention Scoring System-28.

The use of different psychoactive medications was a multivariate significant risk factor (OR 3.34; Figure 2). Detailed observations generated an increased risk with benzodiazepine use (OR 2.89). Patients having an endotracheal or trachea cannula were at greater risk, even after multivariate analysis (OR 8.07). A gastric tube (OR 7.80) and a bladder catheter (OR 5.37) were significant factors after univariate analysis. The risk for the onset of delirium increased with the number of infusions (OR 1.35). Moreover, more than three infusions (2.74) showed a higher risk after multivariate analysis (Figure 2). Patients who were not able to have a regular meal showed a higher risk (OR 3.83) for the development of delirium. Fever before delirium and an arterial catheter could not be identified as a risk factor in this research.

### Factors related to the environment

The isolation of a patient (OR 2.39), with no visible daylight and no visits from relatives (OR 3.73), showed a higher risk of dementia after multivariate analysis (Figure 2 and Table 6). Admittance through the emergency room showed no higher risk for the development of delirium. A transfer from another ward, however, was a significant risk factor (OR 1.98).

**Table 6:** Environmental factors

	n		mean (SD) or %			univariate	multivariate
	D	ND	D	ND	P*	OR (CI)	OR (CI)

admission via emergency room	60/118	119/259	51%	46%	0.22	1.22 (0.79 to 1.88)	

admission via transfer	36/118	47/259	31%	18%	0.006	1.98 (1.20 to 3.28)	

open room in intensive care	52/149	98/359	35%	27%	0.055	1.43 (0.95 to 2.15)	

isolation	16/155	11/368	10%	3%	0.001	3.74 (1.69 to 8.25)	2.89 (1.00 to 8.36)

no visible daylight	70/155	118/368	45%	32%	0.003	1.75 (1.19 to 2.56)	2.39 (1.28 to 4.45)

no clock present or visible	19/155	36/368	12%	10%	0.243	1.29 (0.71 to 2.33)	

number of visitors	88	168	2.4 (1.9)	2.5 (2.0)	0.70	0.97 (0.85 to 1.11)	

no visit	27/96	21/173	28%	12%	0.001	2.83 (1.50 to 5.36)	3.73 (1.75 to 7.93)

physical restraints	25/66	4/226	38%	2%	<0.001	33.84 (11.19 to 102.36)	

Continuous variables are presented in number, mean and standard deviation (SD); categorical variables are presented in number per group and percentage.

* *P *value of difference in groups, calculated with independent samples t-test for continuous variables, with Chi squared for categorical variables.

CI = confidence interval; D = delirium group; ND = non-delirium group; OR = odds ratio.

The use of physical restraints before the onset of delirium showed a very high risk (OR 33.84). The 95% CI (11.19 to 102.36), however, is very wide leaving this factor not appropriate for multivariate analysis.

The absence of a visible clock was no risk factor. Although more delirious patients were admitted in a bed in an open shared room, this factor showed no higher risk (Table 6).

### Multivariate model in the four domains

The significant factors in the different domains were studied using the Nagelkerke R^2^. The significant risk factors in the domain of the patient characteristics were responsible for 20% of delirium. The predisposing cognitive impairment, the only risk factor in the domain of the chronic diseases, was responsible for 2% of delirium. The risk factors in the domain of the acute illness were responsible for 48% of delirium and the fourth domain with factors related to the environment for 53% of delirium.

## Discussion

The overall incidence of delirium in this research was 30%. Risk factors for delirium were divided in four domains: patient characteristics, chronic pathology, acute illness, and environmental factors. Particularly in the latter domains an important number of significant risk factors were identified.

### Factors related to patient characteristics

As in our research, most studies on risk factors for delirium in the intensive care unit did not mention age as a significant factor [7,9]. Research outside the intensive care unit often pointed at the relevant effect of age on the onset of delirium [1,5]. In this specialized unit, the cascade of other risk factors possibly overrules the obvious effect of age. Also, gender had no effect on the development of delirium.

The best-known type of delirium is delirium tremens. The withdrawal of alcohol causes a delirious state. The daily use of three units of alcohol is an important multivariate factor in our study. Alcohol abuse, in the study of Ouimet and colleagues [9], defined as the daily use of more than two units, also shown to be a multivariate risk factor. Therefore, in order to prevent delirium, patients or their relatives must be interviewed as soon as possible to detect daily use of alcohol.

In our research, the risk to develop delirium was elevated after smoking 10 cigarettes each day. Ouimet and colleagues [9] also indicated an effect of active tobacco consumption and Dubois and colleagues [8] calculated a comparable OR after consumption of 20 or more cigarettes each day. The sudden stop in the consumption of nicotine may have caused a withdrawal delirium. Public health data of the World Health Organization revealed that smoking is common in 24% of adults in the USA, 37% in Europe, and 27% in the Belgian population [20]. It might be justifiable to study the effect of nicotine surrogates to prevent delirium in patients with a high consumption of cigarettes. Additionally, patients smoking more than 10 cigarettes are more vulnerable to chronic pulmonary diseases. Lower oxygen saturation in the brain might influence the onset of delirium in these patients.

In our study, patients living alone at home showed a higher risk of developing delirium. This factor possibly interfered with 'no visit before delirium', a significant environmental risk factor. In the group of patients 'not living single at home' 8% did not receive a visit; 28% of patients 'living single at home' did not receive a visit. Further research has to identify the individual effect of this factor.

In our research, neither education nor profession was a risk factor for the onset of delirium.

### Factors related to chronic pathology

This study had a limited approach to factors related to chronic pathology. Research outside the intensive care unit showed possible relations with diabetes, AIDS, or other chronic pathology [5,21].

A previously diagnosed dementia showed to be an important risk factor. Research in the intensive care unit on elderly patients by McNicoll and colleagues [22] found a relative risk of 2.2 (95% CI, 1.0 to 5.0) and by Pisani and colleagues [11] an odds ratio of 6.3 (95% CI, 12.9 to 13.8). Our research, focusing on adult patients, found a similar effect. Patients with an established diagnosis of dementia were at risk of delirium. Advice to screen newly admitted intensive care patients with a dementia screening instrument to detect those who are vulnerable can be given.

### Factors related to acute illness

The factors most studied for a possible relation with the onset of delirium in the intensive care unit are related to either abnormal serum values or the use of psychoactive medication [7-10,23]. The prevalence of the studied abnormal blood values was too small to include in our study.

Psychoactive medication may disturb the neurotransmission in the brain provoking a delirious state. Use of the total group of this medication, either benzodiazepines or morphine, was shown to be a risk factor in this study. As in other research, a more detailed review pointed at the delirious effect of benzodiazepines [8-11]. After the administration of morphine to the patient, the risk for delirium is higher, although not significant. Literature pointed at a higher risk, but only Dubois and colleagues [8] found significant results concerning the use of morphine. The effect of psychoactive medication on the onset of delirium appeals for prudence in the prescription and administration.

Most of the patients were included after a stay of 24 hours in the intensive care unit. Later inclusion in the study was caused by a Glasgow Coma Scale below 10. A longer period where patients did not reach this criterion for inclusion resulted in a higher risk for delirium. Ouimet and colleagues [9] also showed that patients were at higher risk after sedation or coma. Other research pointed to the possible relation between the length of stay in the intensive care unit and the development of delirium [7,24]. The length of stay, however, has been discussed as a time-dependent risk factor or outcome after delirium [9,25,26]. Since most of the patients in this study developed delirium within three days after inclusion, the use of a Cox proportional hazard model, as suggested by Girard and colleagues, did not seem necessary in this research. When studying the length of stay as a risk factor, the clinical relevance of a time-correcting analysis can be questioned. A study on the short-term outcome of delirium can use this method to address the time-dependent bias.

A high risk of mortality at admission indicates a patient with more severe pathology. Although an elevated APACHE II score showed no significant higher risk in our research, as in Dubois and colleagues [8], the combined factor 'higher risk of mortality' showed a significant univariate risk for delirium. In the studies by Pandharipande and colleagues [10] and Ouimet and colleagues [9], this higher risk was significant after multivariate analysis. Similarly, the TISS 28 score, indicating the nursing time needed for each individual patient on a certain day, was related to the onset of delirium. A patient requiring about five hours of nursing care in each shift was at high risk for delirium. Although the interpretation of mortality or severity of illness scores has been discussed for individual patients, higher values indicate a greater illness burden. Patients with these higher scores are at higher risk for delirium. Future research could study cut-off values of risk scores and nursing workload scores as for patients at risk for delirium.

The number of infusions is a significant risk factor in multivariate analysis. It is most likely it is not the infusion itself being linked to the delirious process, but the number of medications administered. This is comparable to the results of Inouye and colleagues [5] in older patients outside the intensive care unit. Also, a treatment with more drugs indicates a more severely ill patient.

Furthermore, many patients in the intensive care unit will not receive normal food, and will have an endotracheal tube, a gastric tube, a bladder or other catheters when necessary for a more invasive treatment. A patient who is more ill will generate more risk factors. Consequently, the cascade of different significant factors in the third domain is related to the degree of illness and consequent treatment. The mentioned factors, however, are often not modifiable due to current pathology and current treatment. Nevertheless, intensive care staff should pay special attention to the removal of tubes and catheters when no longer needed. Prudence with medication is advised and the intensive care staff must pay extra attention to the more severely ill patients.

### Factors related to the environment

As in our study, earlier research did not describe the architectonical structure of the intensive care unit as a possible risk factor. Patients did show a higher incidence of delirium after a stay in an isolation room. The absence of visible daylight, however, is very important. The presence of daylight in the patient's room should be stimulated where possible. This complies with research stating that the disturbance of the circadian rhythm might cause delirium [27,28].

The use of physical restraints was studied before the onset of delirium. Patients were not observed as agitated when restrained before the onset of delirium. The preventive use of soft wrist restraints to protect the position of catheters, tubes, and drains seems to evoke delirium. Likewise, research pointed at a possible relation between restraints and self-extubation [29,30]. The low prevalence of the factor in non-delirious patients impeded interpretation. The high incidence of delirium in patients after physical restraints, however, showed a strong relation. This indicates that the unnecessary use of physical restraints in the intensive care unit must be banned. Inouye and colleagues also showed a higher relative risk of delirium for restrained patients outside the intensive care unit [4]. Further research is needed to study the effect of physical restraints in the onset of delirium.

Admission via the emergency room was not a significant risk factor, whereas the transfer from another ward to the intensive care unit was. The transport of a critical patient is an urgent decision most of the time. This abrupt change of environment seems to influence the onset of delirium.

Patients without visitors were at greater risk of developing delirium. Recent literature pointed at the possible beneficial effects of visitors in the intensive care unit [31]. The prevention of delirium could be an argument in the discussion towards a more open visitor policy.

Visible daylight, where available, and a policy to allow more visits to the patient are factors easy to influence to study the possible beneficial effect on the onset of delirium.

### Domains of factors

The individual effect of a single factor in the onset of delirium is difficult to study. Multivariate analysis excluded many related factors. The cumulative presence of factors always causes a combined effect. Moreover, one factor may cause others. Therefore, the design of a mathematical predictive model based on single factors might not be the best solution. Patients are vulnerable due to patient characteristics or chronic pathology. Multivariate analysis showed the importance of patient characteristics. Additional factors should be studied in the domain of the chronic pathology. The noxious insults, however, are related to the acute illness or environmental factors. The Nagelkerke regression coefficient showed a high prediction of delirium based on the factors in the last domains. Interventions on these noxious insults seems be the best action to prevent delirium.

The use of factor analysis for the assignment of risk factors to different domains could improve the insight in an overall delirium model. Such a model could be useful to consider delirium as the sixth vital sign [32].

### Study limits

The patients included in this study are only a segment of the intensive care population. The inclusion criteria and the selected assessment tool resulted in a subgroup of less sick or recovering intensive care patients. Nevertheless, this research showed that risk factors are also predominant in this specific population.

Not all factors were registered in all participating hospitals reducing the sample size in the joined database, particularly in multivariate analysis. The differences in case mix and the incidence in delirium provided a heterogeneous sample of intensive care patients from the different hospitals. Further research using a more robust method will focus on the differences in the onset of delirium within each hospital.

Factors were assigned to their domain based on experience of different physicians and nurses. Statistical techniques might split factors otherwise. Our model tried to be logical and comprehensive based on known precipitating and predisposing factors.

This study only included an Antwerp population. The results should be confirmed in an international cohort.

## Conclusions

This multicenter study indicated risk factors for delirium in the intensive care unit related to patient characteristics, chronic pathology, acute illness, and environment. Multivariate risk factors were the use of more than three units of alcohol each day, a predisposing cognitive impairment, more than three infusions, an admission for internal medicine, an endotracheal tube or tracheastomy, no visible daylight, isolation, and no visitors. Particularly among those related to the acute illness and environment, several factors are suitable for preventive action.

## Key messages

• Predisposing risk factors for delirium are related to patient characteristics and chronic pathology.

• Precipitating risk factors for delirium are related to acute illness and the environment.

• Several risk factors are suitable for preventive action.

## Abbreviations

APACHE: Acute Physiology And Chronic Health Evaluation; CI: confidence interval; OR: odds ratio; RR: relative risk; SAPS: Simplified Acute Physiology Score; TISS 28: The Therapeutic Intervention Scoring System-28.

## Competing interests

The authors declare that they have no competing interests.

## Authors' contributions

BVR conceived the study with supervision of all authors. ME supervised the statistical analysis. All authors approved the paper after critical reading.
